# The Impact of COVID-19 on the HIV Cascade of Care in Botswana – An Interrupted Time Series

**DOI:** 10.1007/s10461-024-04388-x

**Published:** 2024-06-10

**Authors:** Alice Sehurutshi, Habib Farooqui, Tawanda Chivese

**Affiliations:** 1 Division of Epidemiology and Biostatistics, Stellenbosch University, Cape Town, South Africa; 2 Harvard AIDS Institute Partnership, Gaberone, Botswana; 3 Department of Population Medicine, College of Medicine, QU Health, Qatar University, P.O. Box 2713, Doha, Qatar

**Keywords:** COVID-19, Human immunodeficiency virus (HIV), HIV cascade of care, HIV screening, Sub-saharan africa, Antiretroviral therapy (ART) initiation, Botswana

## Abstract

Globally, Botswana has one of the highest burdens of HIV. This study estimated the impact of the COVID-19 pandemic on the HIV cascade of care in Sub-Saharan Africa. We conducted an interrupted time series analysis on national-level data to estimate the effect of COVID-19 on the numbers of HIV tests, positive HIV tests and ART initiations from April 2019 until March 2021. In multivariable Poisson interrupted time series regression, the COVID-19 lockdown was associated with a 27% decrease in the monthly numbers of HIV tests (IRR 0.73, 95%CI 0.72–0.73), a 25% decrease in HIV positive tests (IRR 0.75, 95%CI 0.71–0.79), and a 43% reduction in ART initiations (IRR 0.57, 95%CI 0.55–0.60). The impact of the pandemic on all three outcomes was worse in males and those aged ≥ 50 years. In conclusion, COVID-19 had a strong negative impact on HIV screening, diagnosis and ART initiation in Botswana.

## Background

Globally, considerable progress has been made to reduce the incidence and impact of the human immunodeficiency virus (HIV) and many people living with HIV (PLWH) are now on lifesaving antiretroviral therapy (ART) and deaths due to HIV have been going down consistently during the past decade [1]. However, the HIV epidemic remains the biggest health challenge for many countries in Sub-Saharan Africa, which currently hosts about two-thirds of the roughly 40 million PLWH globally [1]. The current progress in HIV prevention and treatment has been achieved through a series of evidence-based policies which have targeted each step of the cascade of care for HIV. The HIV cascade of care consists of steps required to achieve viral suppression at an individual level, and control of the epidemic at a population level [2]. These include HIV screening and diagnosis, linkage to care, engagement and retention in care, ART adherence and viral suppression [2, 3]. To prolong the life of PLWH, providing treatment as prevention and progress towards ending the HIV pandemic, the UNAIDS has set out the 95-95-95 goal to be reached by 2030. The 95-95-95 goal aims to diagnose 95% of all PLWH, provide ART to 95% of those diagnosed and achieve viral suppression for 95% of those on treatment [4]. To achieve the 95-95-95 goal, the test and treat strategy should be implemented, i.e., the population at risk is screened for HIV and people living with HIV receive early treatment [5]. If HIV screening and diagnosis are hampered for an extended period, the elements of the care cascade are progressives negatively impacted, thereby increasing the risk of reverting to high HIV incidences, particularly in high-burden countries in Sub-Saharan Africa such as Botswana.

Botswana, one of the countries with a high prevalence of HIV in Sub-Saharan Africa, has made substantial progress towards achieving the 95-95-95 goals. In 2021, 94% of PLWH knew their status, 92% of those living with HIV were receiving ART and 90% of those living with HIV had viral load suppression [6]. Further, Botswana is the first country with a high HIV prevalence that eliminated the mother-to-child transmission of HIV in the year 2021 [6]. However, it is not clear how the provision of HIV services was affected by the COVID-19 health emergency.

Evidence from the few published reports suggests that COVID-19 and the public health measures against it may have impacted the cascade of care for PLWH [7]. In Botswana, the first case of COVID-19 was reported on 30 March 2020 and subsequently, a national 28-day lockdown was imposed on 02 April 2020 [8]. After the lockdown was over, several public health measures aimed at stopping the spread of COVID-19 remained in place, including restrictions on overcrowding, wearing masks and physical distancing together with the limitation of persons in transportation [9]. These public health measures resulted in restricted access to routine healthcare services, except for emergency care [9]. Other initiatives were put in place to minimize these disruptions and ensure basic care for patients continues. In Botswana, PLWH were usually given a one-month supply of antiretrovirals before COVID-19. During the first months of COVID-19, it was allowed for dispensing of 3 months’ supply to ensure adequate patient medication during movement restrictions. This was also implemented in other countries in Sub-Saharan Africa such as Namibia where the strategy included not only an emphasis on multi-month (3–6 months) dispensing of ART but also tracing people living with HIV who missed appointments by telephone to link them back into care [10].

Although there is no evidence yet from Botswana, data from other settings suggest that interventions associated with COVID-19 prevention may have affected the care of PLWH [7, 11]. There is evidence likely that redirecting focus and prioritization of health systems to fight COVID-19 may have had an impact on access to non-COVID-19 diseases [12]. For PLWH, continuity of care could have been further compromised because of the “double” stigma associated with HIV and COVID-19, especially in the early days when there were heightened fears of risk of COVID-19 mortality. COVID-19 disrupted the human resources for HIV care, as nurses, doctors, health care assistants, and laboratory personnel were diverted to care for COVID-19 while the Sir Ketumile Masire Teaching Hospital, became a treating hospital for cases of COVID-19 [9, 13].

This study investigated the impact of COVID-19 on the cascade of care for HIV in Botswana. Specifically, this research compared the number of HIV tests, HIV-positive tests and ART initiations in the 12 months before the first lockdown in Botswana with those in the first 12 months of COVID-19. A further aim of the study was to investigate the effects of COVID-19 on the HIV care cascade in the male and female genders and the different age groups.

## Methods

### Study Design

The study design is an interrupted time series study which used routine data collected nationally by the Botswana Ministry of Health HIV Testing Services and the HIV care program. The interrupted time series was deemed the most appropriate design for the research question because this is the most powerful quasi-experimental design that allows the researchers to assess the impact of a public health intervention at the population level while controlling for time-varying covariates [14]. In this study, the public health intervention that was assessed was the implementation of the COVID-19-related lockdown starting in April 2020 in Botswana.

### Participants

The participants in this study were all adults in Botswana seeking HIV testing and care services.

### Data Sources

National-level data were obtained from the Botswana Ministry of Health HIV Testing Services and the HIV care program for 24 months, 12 months before COVID-19 and the first 12 months during COVID-19. The times from April 2019 until March 2020 and April 2020 until March 2021 were defined as the pre-COVID-19 period and during the COVID-19 period, respectively. The lockdown was implemented in April 2020, and this also signified the start of national public health measures against COVID-19 in Botswana. The following data were collected: time (month), monthly total number of HIV screening tests, monthly total number of individuals with confirmed HIV-positive test results and monthly total number of individuals initiated on ART as well as population estimates during 2019, 2020, and 2021, which were used to standardize the summary data. The population estimates for 2019, 2020 and 2021 are publicly available from Statistics Botswana [15].

### Setting and Sampling Method

All data of eligible participants from the Botswana Ministry of Health- HIV care program and HIV Testing Services during the study months were analyzed, therefore total sampling was used. The age groups included 15–29 years, 30–39 years, 40–49 years, 50–59 years and ≥ 60 years, as captured by the Ministry. Healthcare facilities in Botswana submit monthly data on HIV testing and ART initiations to the Botswana Ministry of Health through a central system, the District Health Information System.

### Outcomes

The main outcomes for the study analysis were the changes in (i) the monthly number of HIV tests, (ii) the monthly number of positive HIV tests, and (iii) the monthly number of ART initiations.

### Data Analysis

The totals, median and interquartile ranges (IQR) for discrete variables such as the number of HIV tests, number of HIV-positive tests, and number of ART initiations were presented during the COVID-19 period. An interrupted time series analysis was undertaken to detect the level and trend change for each outcome, pre-COVID-19 and during COVID-19. Summary data were standardized using the population estimates and regression models used to quantify the effect of COVID-19 public health measures on each of the count outcomes. A multivariable Poisson regression model was used, as the outcomes were count outcomes, to estimate the effect of COVID-19 interventions on the outcomes. The breakpoint for the model was April 2020 which was also the time point for the first national lockdown. From the analysis the following were presented: incident rate ratios (IRR), their 95% confidence intervals (95%CI) and p values. Each model included month as a linear variable, and COVID-19 as an intervention dummy variable and an interaction term between intervention and time to capture level change and trend change. A counterfactual was introduced into the model to assess the outcome in the absence of intervention, and it was assumed that in the absence of COVID-19 the number of HIV tests, number of HIV-positive tests and number of people initiated on ART would remain unchanged. This was represented by a dotted line in the scatter plot for each outcome. STATA-16 was used for the analysis.

### Ethical Considerations

The study received approval from the Botswana Ministry of Health: Health Research and Development Committee (HRDC), reference number: HPDME 13/18/1 and HPDME 6/13/1 as well as the Stellenbosch University Health Research Ethics Committee (SUHREC), reference number: S22/03/005_COVID-19. A waiver of informed consent was requested from the ethics committees since the data collected were not at the individual/participant level and de-identified. Permission to access and use the data was received from the Botswana Ministry of Health Department of Health Services Management.

## Results

A total of 416,201 HIV screening tests were done nationally in the 12 months during the pre-COVID-19 period while only 286,376 HIV tests were done in the 12 months during COVID-19. Compared to the pre-COVID-19 period, there were large declines in all three outcomes, during COVID-19; the total numbers of HIV tests declined by 31.2%, the total number of HIV-positive test results fell by 30.7% and the total number of individuals initiated on ART was reduced by 35.2% (Table 1). The declines were higher in males for all three outcomes, with total HIV tests declining by 46%, total HIV-positive test results declining by 35% and total ART initiations declining by 39% (Table 1). In females, the total tests, positive results and initiations also declined but not at the rate that was observed in males (Table 1). Although reductions were observed in all age groups, the older age groups (50–59 years and ≥ 60 years) showed the highest reductions in the two outcomes where data were available: HIV tests and HIV-positive test results (Table 1). Data on ART initiations within age groups were not available.

**Table 1:** Total number of HIV tests, HIV positive tests and ART initiations by COVID-19 period

	Total HIV tests			Total HIV Positive tests			Total ART initiations		
	Before COVID-19	During COVID-19	% change	Before COVID-19	During COVID-19	% change	Before COVID-19	During COVID-19	% change
Totals	416 201	286 376	-31.2	13 808	9 565	-30.7	15 939	10 324	-35.2
Male	151 935	82 861	-45.5	5 814	3 800	-34.6	6 761	4 141	-38.8
Female	264 266	203 515	-23	7 994	5 765	-27.9	9 178	6 183	-32.6
15–29 years	221 463	160 144	-27.7	4 563	3 195	-30			
30–39 years	129 047	90 043	-30.2	5 324	3 638	-31.7			
40–49 years	45 206	28 427	-37.1	2 909	2 053	-29.4			
50–59 years	20 485	7 762	-62.1	1 012	679	-32.9			
≥ 60 years	18 077	9 418	-47.9	476	214	-55			

NB – data on initiations on ART by age group were not available. Percentage change was calculated as (tests during COVID-19 – tests before COVID-19)/ tests before COVID-19

### THE Effect of COVID-19 on the Number of HIV Tests

**Fig. 1:**
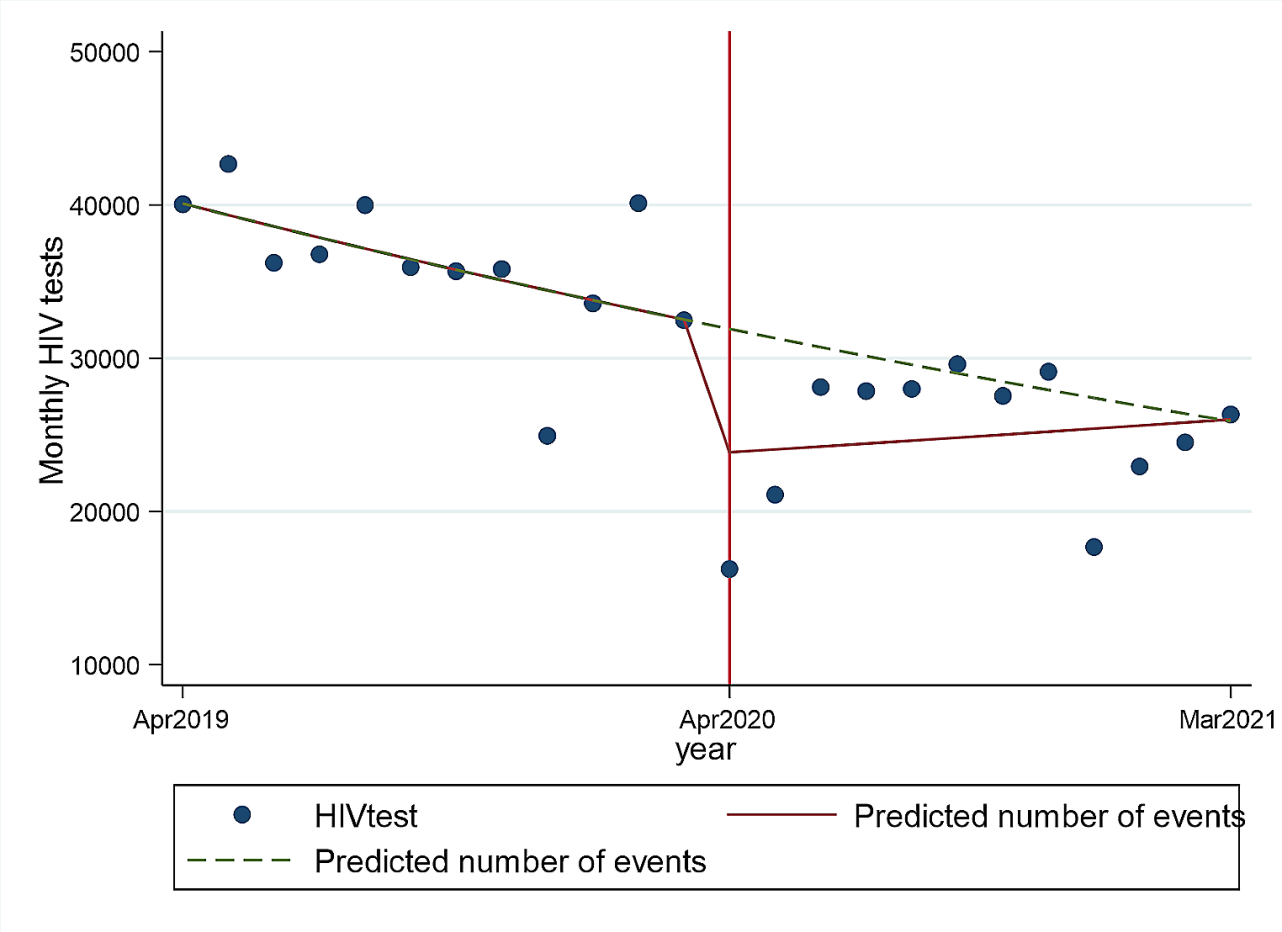
Fitted values of monthly HIV tests by COVID-19 period. NB: The solid line represents the number of HIV test results observed and the dotted line shows what could have been expected in the absence of COVID-19

In the 12 months during COVID-19, compared to the period before COVID-19, there were a significantly lower median number of HIV tests per month (36,081.5, IQR 34,630 − 40,021.5 vs. 26,932, IQR 22,015–28,056, respectively, *p* < 0.001) (Table 2). After multivariable Poisson regression, the estimated immediate effect of the COVID-19 lockdown was a 27% decrease in the mean monthly number of HIV tests (IRR 0.73, 95%Cl 0.72–0.73) (Table 3, Fig. 1). The number of monthly HIV tests remained low in the 12-month period after the lockdown (Table 3, Fig. 1).

**Table 2:** Monthly numbers of HIV tests, HIV positive tests and ART initiations by COVID-19 period

	HIV test (Monthly median (IQR))			HIV positive tests (Monthly median (IQR))			ART initiations (Monthly median (IQR))		
	Before COVID-19	During COVID-19	Mann Whitney U Test statistic (P value)	Before COVID-19	During COVID-19	Mann Whitney U Test statistic (P value)	Before COVID-19	During COVID-19	Mann Whitney U Test statistic (P value)
Overall	36 081.5 (IQR 34 630 − 40 021.5)	26 932 (IQR 22 015–28 056)	z = 5.459 (*p* = 0.0013)	1 205 (IQR 1 129 -1 280)	854 (IQR 800.5-877.5)	Z = 3.638 (*p* < 0.001)	1 235 (IQR 1 090 − 1 400)	830.5 (IQR 815-938.5)	Z = 4.157 (*p* < 0.001)
Gender									
Female	21 884.5 (IQR 21 115.5–24 269.5)	18 301.5 (IQR 15 318.5–19 042)	z = 3.695 (*p* < 0.001)	687 (IQR 637–714)	504 (IQR 464–519)	z = 3.580 (*p* = 0.0001)	708 (IQR 646–815)	500 (IQR 479–549)	< z = 4.041 (*p* = 0.001)
Male	13 114 (IQR 11 897–13 883.5)	7 606.5 (IQR 5 847.5–7 910.5)	Z = 4.041 (*p* < 0.001)	473 (IQR 438–541)	323 (IQR 289–348)	z = 4.041 (*p* < 0.001)	510 (IQR 460–612)	339 (IQR 316–381)	Z = 4.157 (*p* < 0.001)
Age groups									
15–29 years	8 069 (IQR 5 571–13 122)	5 541 (IQR 3 132 − 11 342)	Z = 2.763 (*p* = 0.005)	154 (IQR 97–280)	112 (IQR 64–209)	z = 2.773 (*p* = 0.0049)	NA	NA	
30–39 years	5 203 (IQR 4 587-6 218)	3 328 (IQR 2 767- 5 219)	Z= 3.588 (*p* = 0.0002)	229 (IQR 192–253)	147 (IQR 130–174)	Z = 4.929 (*p* < 0.001)	NA	NA	
40–49 years	1 988 (IQR 1 684-2 039)	1 278 (IQR 1 012 − 1 334)	Z = 5.444 (*p* < 0.001)	123 (IQR 103–137)	86 (IQR 77–96)	Z = 4.466 (*p* < 0.001)	NA	NA	
50–59 years	874 (IQR 724–957)	267 (IQR 212–470)	Z = 5.877 (*p* < 0.001)	39 (IQR 36–50)	30 (IQR 22–34)	Z = 4.584 (*p* < 0.001)	NA	NA	
≥ 60 years	715 (IQR 641–875)	394 (IQR 319–448)	Z = 5.815 (*p* < 0.001)	20 (IQR 15–22)	10 (IQR 7–10)	Z = 5.459 (*p* < 0.001)	NA	NA	

NB – data on initiations of ART by age group were not available

**Table 3:** Incidence rate ratios (IRR) for the effect of COVID-19 on HIV tests, HIV positive tests and ART initiations from multivariable Poisson regression

	HIV tests IRR (95%CI)			HIV positive tests IRR (95%CI)			ART initiations IRR* (95%CI)		
	Pre-lockdown trend	During COVID-19	Post-lockdown trend	Pre-lockdown trend	During COVID-19	Post-lockdown trend	Pre-lockdown trend	During COVID-19	Post-lockdown trend
Overall	0.98 (0.98–0.98)	0.73 (0.72–0.73)	1.03 (1.03–1.03)	0.98 (0.97–0.98)	0.75 (0.71–0.79)	1.03 (1.02–1.03)	1.04 (1.04–1.05)	0.57 (0.55–0.60)	0.94 (0.94–0.95)
Female	0.99 (0.99–0.99)	0.83 (0.82–0.84)	1.01 (1.01–1.01)	0.98 (0.97–0.98)	0.84 (0.78–0.90)	1.02 (1.01–1.03)	1.04 (1.04–1.05)	0.60 (0.57–0.64)	0.94 (0.93–0.95)
Male	0.97 (0.97–0.97)	0.53 (0.52–0.54)	1.06 (1.05–1.06)	0.99 (0.98–0.99)	0.64 (0.59–0.70)	1.03 (1.02–1.04)	1.04 (1.04–1.05)	0.53 (0.49–0.58)	0.94 (0.93–0.95)
15–29 years	0.98 (0.98–0.98)	0.78 (0.77 − 0.79)	1.02 (1.02–1.02)	0.98 (0.97–0.98)	0.77 (0.70 − 0.85)	1.03 (1.02–1.04)			
30–39 years	0.98 (0.98–0.98)	0.73 (0.71–0.74)	1.03 (1.02–1.03)	0.98 (0.97–0.99)	0.75 (0.69 − 0.82)	1.03 (1.02–1.04)			
40–49 years	0.98 (0.98–0.98)	0.60 (0.58–0.62)	1.04 (1.04–1.05)	0.99 (0.98–1.00)	0.74 (0.66–0.83)	1.01 (1.00-1.03)			
50–59 years	0.97 (0.96–0.97)	0.41 (0.38 − 0.43)	1.06 (1.05–1.06)	0.97 (0.96–0.99)	0.74 (0.61 − 0.91)	1.03 (1.00–1.06)			
≥ 60 years	0.97 (0.97–0.98)	0.57 (0.54 − 0.60)	1.04 (1.03–1.05)	0.97 (0.94–0.99)	0.58 (0.41 − 0.81)	1.03 (0.98–1.08)			

NB: A multivariable Poisson regression model was used, as the outcomes were counts, to estimate the effect of COVID-19 interventions on the outcomes, number of HIV tests, HIV positive tests and ART initiations. The breakpoint for the model was April 2020 which was also the time point for the first national lockdown and when public measures were implemented. From the analysis the following were presented: incident rate ratios (IRR), their 95% confidence intervals (95%CI) and p values. Each model included month as a linear variable and COVID-19 as an intervention dummy variable. A counterfactual was introduced into the model to assess the outcome in the absence of COVID-19 and it was assumed that in the absence of COVID-19 the number of HIV tests, number of HIV positive tests and number of people initiated on ART would remain unchanged

*Data on initiations by age group were not available

The impact of COVID-19 lockdown on the number of HIV tests was larger in males, with multivariable Poisson regression showing a 47% reduction in the mean monthly number of HIV tests (IRR 0.53, 95%Cl 0.52–0.54) (Table 3). In females, COVID-19 lockdown was associated with a 17% decrease in the mean monthly number of HIV tests (IRR 0.83, 95%Cl 0.82–0.84) (Table 3). The multivariable Poisson regression showed that COVID-19 impacted the number of tests across all age groups, but the biggest impact was observed in the older age groups, with the highest impact in the 50–59 years, with a 59% reduction in HIV tests (IRR 0.41, 95%CI 0.38–0.43, *p* < 0.001) (Table 3).

### The Effect of COVID-19 on the Number of HIV-Positive Tests

**Fig. 2:**
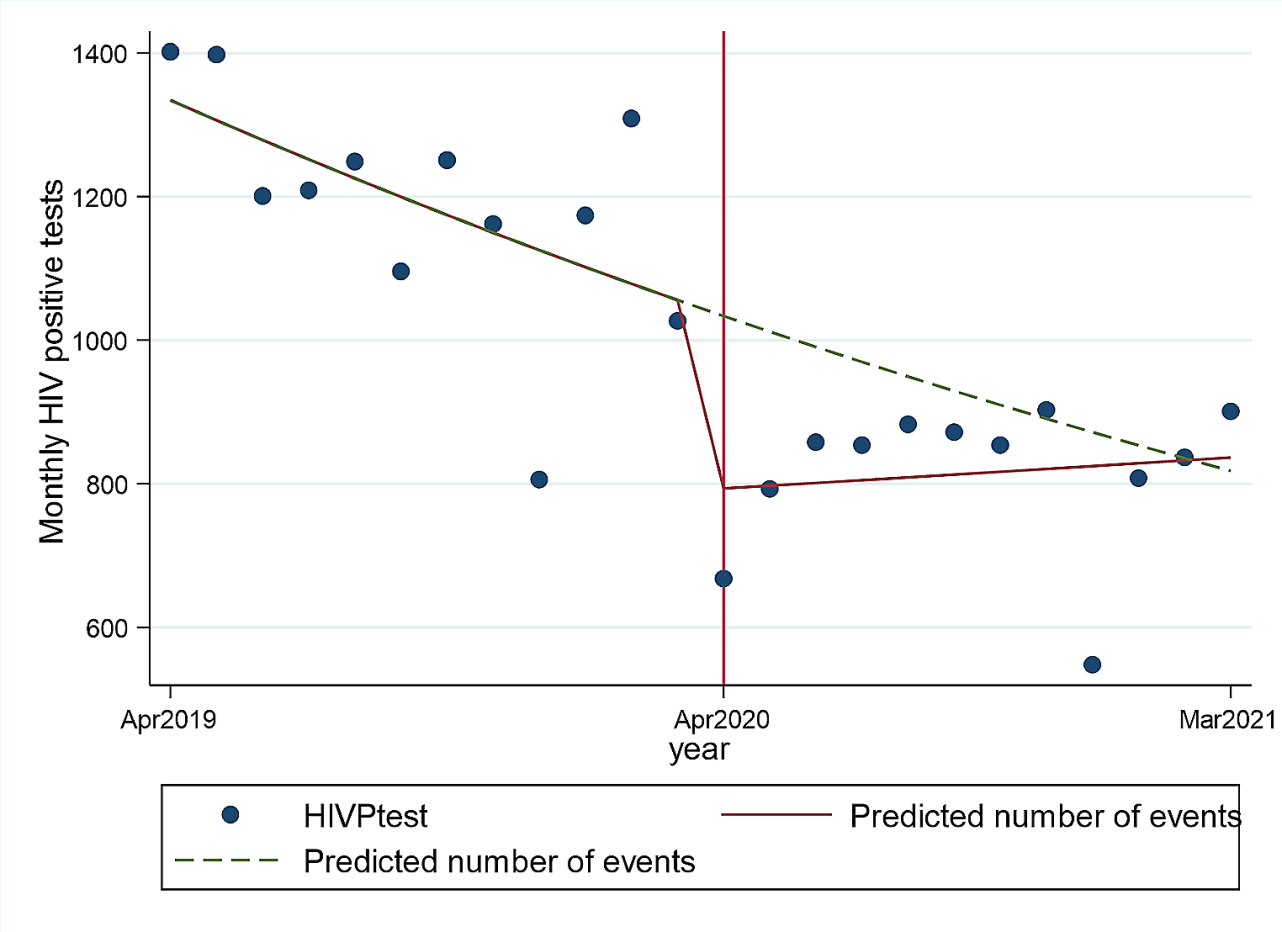
Fitted values of monthly HIV-positive tests by COVID-19 period. NB: The solid line represents the number of HIV positive tests and the dotted line (counterfactual) shows what could have been expected in the absence of COVID-19

In the 12 months during COVID-19, compared to the period before COVID-19, there were significantly lower median HIV-positive tests (1205, IQR 1129–1280 vs. 854, IQR 800.5-877.5: *p* < 0.001) (Table 2). After multivariable Poisson regression, overall, the COVID-19 lockdown resulted in a 25% decrease in mean monthly HIV-positive test results (IRR 0.75, 95%Cl 0.71–0.89) (Table 3, Fig. 2). Furthermore, in the 12-month post-lockdown period, the number of HIV-positive tests remained lower than the corresponding pre-lockdown period (Table 3, Fig. 2).

Similar to the pattern observed for the numbers of HIV tests, the effect of COVID-19 was more pronounced in males, with the multivariable Poisson regression showing a 36% reduction in the mean monthly number of HIV-positive tests in males at lockdown(IRR 0.64, 95%Cl 0.59–0.70) and a 16% decrease in the mean monthly numbers of HIV positive tests in females (IRR 0.84, 95%Cl 0.78–0.90), again with minimal recovery in the post-lockdown period (Table 3). In the age groups, the effect of COVID-19 on the number of HIV-positive tests again showed a gradual worsening in the older age groups, with the greatest reduction being observed in the ≥ 60-year-old age group (Table 3). In both genders and in all age groups, in the 12-month post-lockdown period, the number of HIV-positive tests remained lower than the 12 months before the lockdown (Table 3).

### The Effect of COVID-19 on the Number of ART Initiations

**Fig. 3:**
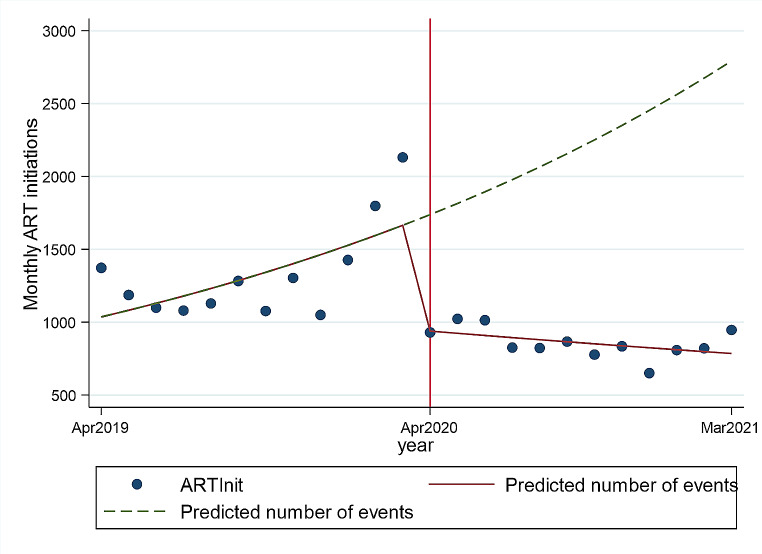
Fitted values of monthly overall ART initiations by COVID-19 period. NB: The solid line represents the number of ART initiations, and the dotted line (counterfactual) shows what could have been expected in the absence of COVID-19.

In the 12 months during COVID-19, compared to the period before COVID-19, there was a significantly lower median number on ART initiation (1235, IQR 1090–1400 vs. 830.5, IQR 815-938.5: *p* < 0.001) (Table 2). In multivariable Poisson regression, the COVID-19 lockdown resulted in an estimated 43% decrease in the mean monthly number of ART initiations (IRR 0.57, 95%Cl 0.55–0.60), (Table 3, Fig. 3). The multivariable Poisson regression showed a larger impact of the COVID-19 lockdown on ART initiations in males with a 47% estimated decrease in mean monthly ART initiations (IRR 0.53, 95%CI 0.49–0.58) while mean monthly ART initiations went down by 40% in females (IRR 0.60, 95%CI 0.57–0.64). The number of monthly ART initiations remained low during the 12 months after the lockdown, overall (Table 3 Fig. 3) and for both genders (Table 3).

## Discussion

In this interrupted time series using nationwide programmatic data, we found that COVID-19 disrupted the cascade of HIV care in Botswana and resulted in substantial reductions in HIV screening tests, the detection of HIV-positive cases and ART initiations during the first 12 months of the pandemic. The effect of COVID-19 on these components of the HIV cascade of care was more pronounced in males and in age groups above 50 years.

We found that the mean monthly number of HIV screening tests decreased by almost one-third during the 12 months after the first lockdown was implemented, in the whole population, with higher decreases observed in males and individuals in age groups above 50 years. Dorward et al. noted a similar trend in 65 primary care clinics in KwaZulu-Natal, South Africa, although with a shorter time measurement [11]. Their analysis showed a 48% decrease in HIV testing in the first month of lockdown in April 2020 [11]. Rick et al. also observed a reduction between 26% and 45% in the number of HIV tests in the period from January to August 2020 compared to the same pre-pandemic period in 2019 in 44 countries across four continents, with a larger impact being observed in Latin America [16]. A decrease in the number of HIV tests done will lead to more people living with HIV not knowing their status as well as a delay in initiating ART, and consequent increases in new cases and late HIV diagnoses.

Our findings suggest that the number of HIV-positive tests decreased by a quarter after the implementation of the lockdown, compared to the 12 months before COVID-19. The decreases were worse in males and the age groups older than 50 years, similar to what was observed in the number of HIV tests. This reduction in the number of HIV-positive tests could have been most likely a consequence of low testing rates during the period. This trend has also been described in other countries. In Uganda, which had social distancing policies instituted on March 12, 2020, HIV-positive tests declined by 75% in the first 2 weeks of April [12]. Similarly, in South Africa, during the level-5 lockdown, a 22% reduction in average weekly HIV-1 viral load testing and a 33% reduction in CD4 cell count testing done by the National Health Laboratory Service was noted in comparison to the pre-lockdown periods [17]. Similar reductions were also noted in the KwaZulu Natal province of South Africa [11]. However, data from some countries showed slight increases in the number of HIV-positive tests during the early part of 2020 compared to 2019 (3.73% and 3.65% respectively), perhaps because the reporting of positive cases tends to lag behind the total tests. Overall, the evidence suggests that COVID-19 had a significant negative impact on the detection of positive cases in sub-Saharan Africa, and the implications are similar to those of reduced testing, i.e., lower case detection will lead to a higher incidence and late presentation for care for PLWH.

In the current study, ART initiations were the most affected component of the cascade of care with a decrease of 49% after the lockdown was implemented. These findings are largely similar to findings from South Africa where a decrease of 46% in weekly ART initiations was observed [11], and in four other countries in Africa, namely Tanzania, Kenya, Uganda and Nigeria [18]. In St. Louis, during the period April 2020 to May 2020, 13.5% of PLWH reported limited access or lost access to medical care during the COVID-19 pandemic compared to 3.7% of participants who were HIV negative [19]. In Botswana, similar to many other countries, ART initiations were usually done through in-person clinic visits and restrictions in movement would predictably have negatively impacted these visits.

The interruptions in the HIV cascade of care that we have noted in Botswana, a country with one of the most robust HIV screening and care programs in Africa, may potentially reverse the progress that has been made in the fight against HIV. For PLWH, continuity of care could have been further compromised because of the “double” stigma associated with HIV and COVID-19, especially in the early days when there were heightened fears of risk of COVID-19 mortality. A modelling study suggested that a 6-month interruption of supply of ART drugs across 50% of the population of PLWH who are on treatment would be expected to lead to a 60% increase in HIV-related deaths over a 1-year period compared with no disruption [20]. There is therefore a need to ensure continued care for people living with HIV during health emergencies [21, 22]. In Victoria, Australia, people living with HIV were able to access their HIV provider during the pandemic, assisted by telehealth, and therefore access to HIV care and ART was largely uninterrupted [23]. In the USA, barriers to access to care such as transportation costs, participant work hours, and household obligations were reduced by the convenience of the Virtual Visit feature [24]. However, these technology features have also come with challenges where users need to comprehend their use [24, 25] and in low-to-middle income countries such as the ones in Sub-Saharan Africa, a lack of resources, poor connectivity and low affordability impede their implementation. Other methods such as mobile clinics and/or pharmacies for ART initiations and HIV self-testing may reduce the impact of restricted movement and access to healthcare facilities [26].

Notably, the effect of COVID-19 was more on ART initiations compared to its effect on both the number of tests and the number of test-positives. First, the absolute numbers of ART initiations were always lower than the numbers testing positive, and this is likely because ART initiation depends on individuals testing positive being referred and initiated, a process that usually takes time. Second, the effect of COVID was higher in ART initiations (IRR of 0.57 overall) compared to its effect on the number of tests (IRR of 0.73) and number of test-positives (IRR of 0.75). A possible explanation for this difference in effect could be that ART initiation requires more patient contact time with the health system, something that the lockdown and restrictive measures would have had a big impact on. Before patients are initiated on ART, several tests are needed including viral load measurements, CD4 counts, kidney and liver function for better treatment outcomes and monitoring [28].

One of the major strengths of this study is the use of an interrupted time-series research design to estimate the impact of public health measures on the cascade of care for the HIV care program in Botswana. This study also provides a comprehensive analysis using data from the national program stratified by age and gender. However, some limitations of the current study include the lack of data from private and non-profit healthcare providers, although this is mitigated by the fact that most HIV care is provided through state-funded healthcare providers in Botswana. Additionally, we did not stratify the analysis by socioeconomic group, education, or location due to a lack of relevant data. Furthermore, we did not have access to data on viral load, morbidity and mortality, which prevented us from investigating their impact. Finally, the quality of data used in the current study may have been affected by documented data quality issues, although a roadmap for improving data quality was instituted since 2018 and may have improved the quality of the current data [27].

## Conclusion

In Botswana, COVID-19 negatively impacted HIV screening, the detection of HIV-positive cases and ART initiations. This impact is likely to have also occurred in other HIV high-burden countries in Sub-Saharan Africa. These findings suggest a need to protect key existing health services in times of public health emergencies.
